# Primary intrarenal teratoma in an adult: A case report and review of literature

**DOI:** 10.4103/0970-1591.56184

**Published:** 2009

**Authors:** T. J. Nirmal, S. Krishnamoorthy, A. Korula

**Affiliations:** Department of Urology, Christian Medical College, Vellore, India; 1 Department of Pathology, Christian Medical College, Vellore, India

**Keywords:** Teratoma, extragonadal, kidney

## Abstract

A 35-year-old male presented with left loin pain. On evaluation, he was diagnosed to have a left renal lower polar mass. He underwent partial nephrectomy. The histopathological examination was suggestive of teratoma of the kidney. We present this case, as intrarenal teratomas in adults are extremely rare and only a very few cases are reported in literature.

## INTRODUCTION

Extragonadal germ cell neoplasms are predominantly located in the retroperitoneum. Germ cell neoplasms arising from the kidneys are rare. Primary intrarenal teratomas are extremely rare. Majority of the renal teratomas are known to occur in childhood; even more uncommon is their presentation in adults. We present a case of primary renal teratoma in a 35-year-old male.

## CASE REPORT

**Figure 1:**
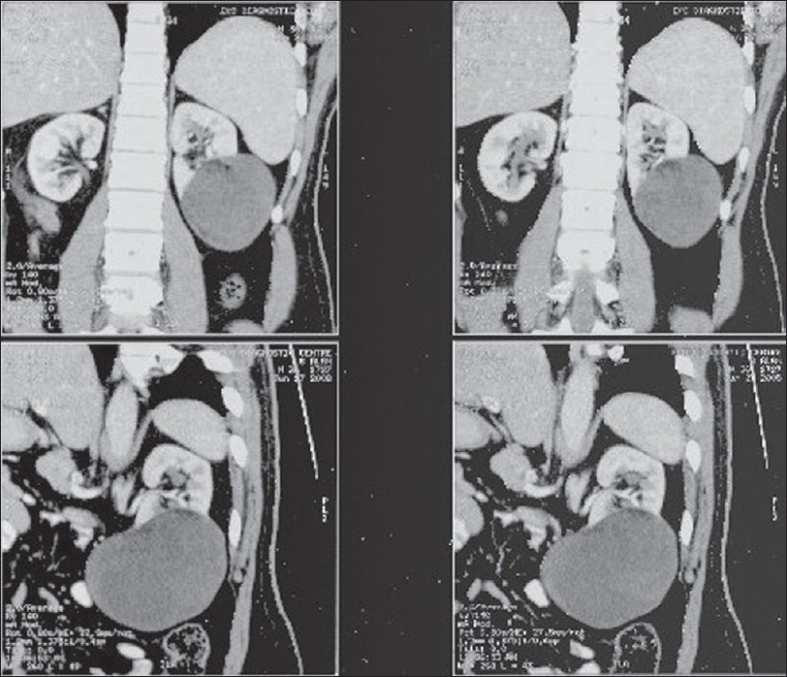
Contrast enhanced CT scan showing well-defined homogenously hypodense mass arising from the lower pole of the left kidney

A 35-year-old male presented with dull aching left loin pain of six months duration. The pain was intermittent and nonradiating. He had no lower urinary tract symptoms, hematuria, or fever. Physical examination revealed a normal external genitalia and testes. His hemoglobin level, ESR, and urine microscopy were within normal limits. Liver function tests and chest X-ray were also normal. Contrast enhanced computed tomography revealed a 12 × 9 cm well-defined, homogenously hypodense mass arising from the lower pole of the left kidney. The mass was confined to the kidney and there was no significant perihilar lymphadenopathy [Figure 1].

Intraoperatively, a 12 × 9 cm soft, yellowish mass was seen arising from the lower pole of the left kidney. The mass was excised completely with a thin rim of normal renal parenchyma.

### Histopathology

**Figure 2:**
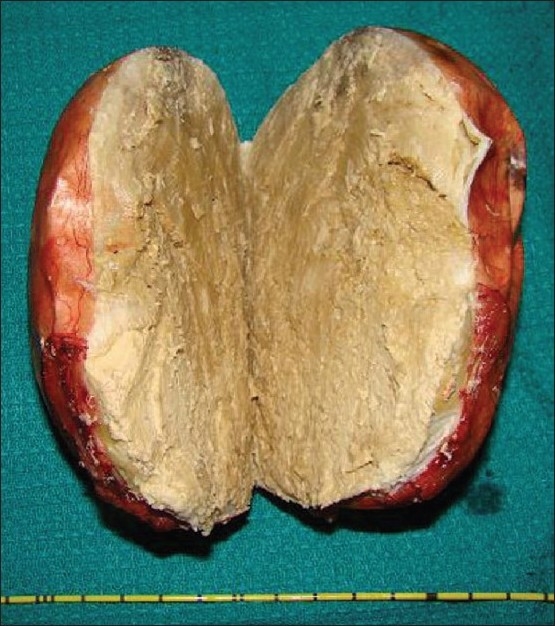
A 12 × 9 × 9 cm cystic mass with whitish, tan and pultaceous material on cut section

Gross examination showed an ovoid cystic mass of 12 × 9 × 9 cm and weighing about 750 g. Cut section revealed whitish, tan and pultaceous material [Figure 2].

**Figure 3:**
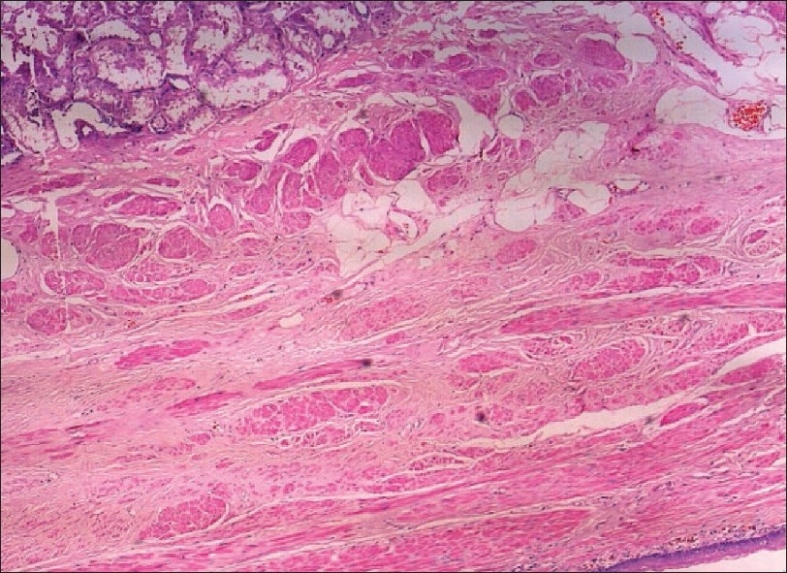
Renal parenchyma in the top left with wall of the teratoma below (H and E, ×50)

**Figure 4:**
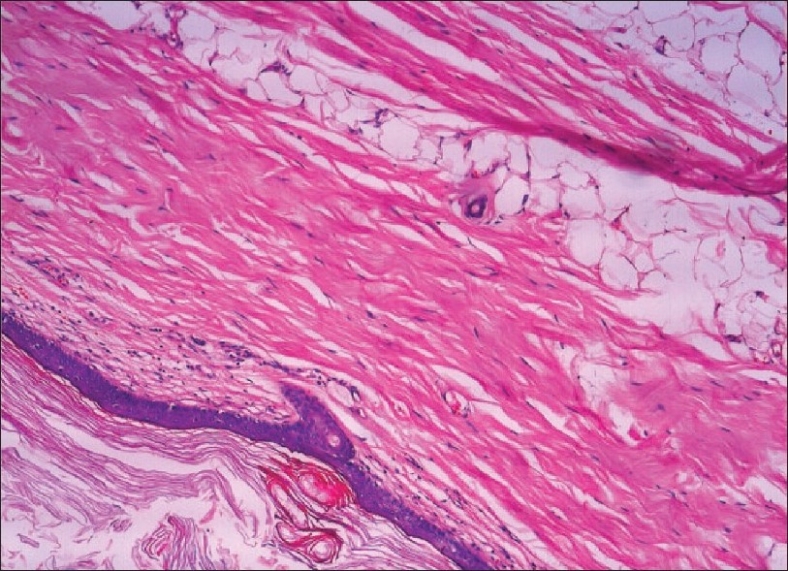
Teratoma lined by squamous epithelium with keratin in the lumen and sebaceous glands with surrounding adipose tissue smooth muscle in the wall (H and E, ×100)

Microscopy revealed renal parenchyma with a cystic lesion lined by stratified squamous epithelium with lamellated keratin in the lumen. Sebaceous glands were seen in the lining epithelium with surrounding adipose tissue smooth muscle. The adjacent renal parenchyma showed tubular atrophy, interstitial fibrosis, and glomerular atrophy with focal glomerulosclerosis. A few dysplastic tubules were seen in the medulla set in a fibrotic stroma [Figures 3 and 4].

## DISCUSSION

Teratomas are neoplasms of embryonal origin. They are predominantly extragonadal, occur in the midline and are generally associated with reproductive organs.[1] Extragonadal teratomas are more commonly seen in the retroperitoneum and mediastinum. In infants, sacrococcygeal region is more commonly involved. Retroperitoneal teratomas exhibit a bimodal presentation, with peaks in the first six months of life and early adulthood.[2] Kidney is one of the least common locations for teratomas and other germ cell tumors.[3] The first reported case of teratoma of kidney was in 1934, when Mc Curdy described this entity in a seven-week-old child with Prune-Belly syndrome.[4] Singer and Anders had reported a case of primary teratoma of the kidney in a two-month-old boy who presented with a palpable flank mass.[5]

So far, only about 23 cases of renal teratoma have been described in literature.[6] Majority of these cases could either be a renal extension of retroperitoneal teratoma or Wilm's tumor with teratoid features.[7] However, they must be considered in the differential diagnosis for abdominal masses in both children as well as adults. For a diagnosis of teratoma to be made, it is important for the pathologist to exclude two major differential diagnoses: (i) metastasis from a gonadal primary tumor and (ii) glomerular and tubular differentiation of nephroblastoma (Wilm's tumor).[6]

Teratomas are generally solid and avascular but when cystic, may sometimes be confused with cystic lesions of the kidney. Otani et al. reported a case of a six-year-old boy with intrarenal cystic teratoma, associated with renal dysplasia.[8] Kojiro et al. described the first case in an adult of 40 years in 1976.[9] Majority in children have a benign clinical course; however, it is difficult to assess the natural history of teratoma occurring in adults because, metastasis can occur even in well-differentiated teratomas of other organs and can occur the same way in kidneys as well.[10] This leads to worsening of prognosis and hence complete excision is totally justified. The purpose of this review was to stress on the fact that though primary renal teratomas in adults are extremely rare, this entity must be taken into consideration in the differential diagnosis of any renal mass in adults as well.
